# Design and evaluation of a coalition-led obesity initiative to promote healthy eating and physical activity in low-income, ethnically diverse communities: the Go! Austin/Vamos! Austin initiative

**DOI:** 10.1186/s13690-019-0350-4

**Published:** 2019-05-29

**Authors:** Alexandra van den Berg, Aida Nielsen, Nika Akhavan, Carmen Llanes Pulido, Semonti Basu, Aliya Hussaini, Christine Jovanovic, Kathryn Janda, Laurence Denis, Nalini Ranjit

**Affiliations:** 1 The University of Texas Health Science Center at Houston, School of Public Health in Austin, 1616 Guadalupe Street, Suite 6.300, Austin, TX 78701 USA; 2 Go! Austin/Vamos! Austin, 3710 Cedar Street Suite #230, Austin, TX 78705 USA; 3 Michael and Susan Dell Foundation, PO Box 163867, Austin, TX 78716 USA

**Keywords:** Community-based initiative, Low-income communities, Program evaluation, Physical activity, Access to healthy food

## Abstract

**Background:**

Go! Austin/Vamos! Austin (GAVA) is a coalition-led health initiative that targets low-income communities with disparities in access to healthy food and physical activity*.* The purpose of this initiative was to increase healthy eating and physical activity among residents by facilitating access to food and physical activity opportunities through environmental and policy changes. Although GAVA is ongoing, this paper describes the original GAVA intervention and the 5-year evaluation study (2013–2018), presenting selected baseline data obtained through its cohort sub-study.

**Methods:**

To assess the impact of GAVA, the evaluation plan included multiple sub-studies and involved collection of quantitative, qualitative, and observational data at different levels. The main cohort sub-study followed 313 parent-child dyads over 5 years. Annually, parents completed self-administered surveys regarding awareness and use of community assets/resources as well as their diet and activities. Heights and weights also were measured.

**Results:**

Cohort participants were primarily Hispanic (87%), very low-income (77%), and food insecure (58%), with high overweight/obesity prevalence among both parents (81%) and children (41%). Awareness and use of community physical activity and healthy eating resources were low, and reported barriers to using these resources were many. Engagement in physical activity and healthy eating also was low.

**Conclusions:**

Given the baseline statistics, GAVA resident teams chose and implemented strategies to address the noted barriers and low usage of community resources. This approach built community capacity and governance. Both the GAVA intervention approach and evaluation protocol can serve as models for other community initiatives to be implemented in other locations and contexts.

## Background

Although some leveling of obesity rates has been noted in recent years, the prevalence of obesity among adults and children in the United States (U.S.) remains high and is accompanied by associated increases in the incidence of Type 2 diabetes, cardiovascular diseases, hypertension, and various forms of cancer [1–3]. On average, 34.9% of adults in the U.S. are considered obese; however, the health burden of obesity falls disproportionately on individuals who live in low-income or minority households [1–3]. In Texas, the overall prevalence of obesity is 32.4%, while among Hispanics individuals in Texas, the prevalence is 38.8% [4].

Obesity is a complex health issue with many causes, with dietary and physical activity behaviors as two key contributors [5]. Research indicates that low-income individuals are less likely to engage in healthy eating and physical activity behaviors than are higher-income populations [6, 7]. The causes of these disparities are multifactorial, but an important influence on low-income individuals’ eating and physical activity behaviors is the community environment in which they live [8, 9]. Low-income families tend to live in communities that are more likely to have inadequate access to healthy food but greater access to unhealthy food, and are less likely to have access to safe, high-quality physical activity opportunities [9, 10]. These types of underserved communities are often referred to as *food deserts* or *food swamps*, as they tend to have fewer food retailers who sell healthier food products and more food retailers who sell unhealthy foods [11–13]. Food desert-type communities are often also *transit deserts* due to a limited infrastructure for active travel and often lack safe places to be physically active, such as parks and trails [14, 15]. Therefore, interventions that target obesity need to be multi-level and include “up-stream” strategies that improve the built nutrition and physical activity environments.

Among the population-based interventions described in the literature, few have targeted minority populations, specifically, and few include comprehensive and robust evaluation results. A 2004 review of a population-based intervention that targeted healthy eating and active living among non-white communities found only 23 ethnically-inclusive intervention studies over the previous 30 years [16]. Of the 23 studies, fewer than half presented outcome evaluation data. Since then, other ethnically inclusive population-based studies have been conducted, but only a few have included strong evaluations or outcome data [17].

Evaluation of comprehensive place-based interventions is difficult due to the complex, multifaceted, and dynamic nature of these interventions [18]. Intervention strategies that were specified initially may not be relevant or appropriate in the community, and need to be evolving based on community needs and concerns that may result from changing demographics and environmental factors. Evaluation of these interventions thus needs to be ongoing as a means to provide continual feedback to the implementers. The Go! Austin/Vamos! Austin (GAVA) initiative was created as a place-based, multi-component obesity intervention designed to improve the built environment to increase access to healthy food and physical activity opportunities (and is described in further detail elsewhere) [19]. GAVA is unique among community based interventions in many ways. GAVA, from the outset, has allowed residents leeway in choosing strategies to implement for neighborhood improvement. While this implementation strategy has greater replicability and sustainability over the long term [19], it does not easily allow the deployment of standardized, synchronized and targeted (e.g., age-stratified) intervention strategies that have been shown to be successful in other interventions [20–22]. Given the challenges of evaluating this initiative with a relatively low dose of intervention, it is imperative to develop a comprehensive evaluation plan that uses a mixed methodology. The purpose of this paper is to (1) describe the GAVA evaluation plan with a focus on the cohort sub-study and (2) present selected baseline demographic, perceptual, and behavioral data obtained through the cohort sub-study.

## Methods

The overarching goal of the 5-year GAVA evaluation study was to measure the impact of the GAVA initiative on awareness of resources, barriers to using resources, utilization of resources, obesity-related behaviors, and weight status of residents in a low-income, ethnically diverse community. To allow for a comprehensive evaluation of the impact of the GAVA intervention, the evaluation protocol involved multiple sub-studies, including a 5-year Cohort Study of parent-child dyads, a Community Readiness Study, and a Resources Assessment Study (e.g., parks, recreation center, corner stores, community gardens). Each of the studies used its own recruitment strategy and study design and involved different types of data (Table 1). All procedures were approved by the University of Texas Health Science Center’s Institutional Review Board (HSC-SPH-13-0107; HSC-SPH-13-0108) and the appropriate school district review boards. All materials were available both in English and Spanish and were tested with members of the target population before use in the field.

**Table 1:** Overview of GAVA’s sub-studies

Sub-Studies	Purpose	Details	Frequency of data collection	Number of participants at baseline	Type of data	Study design	Source of instrument
Cohort Study	To compare outcome variables of families living in intervention versus control communities	Parents self-report survey for self, and measured child and adult heights and weights. Cohort started when child was in Kindergarten	Annual for 5 years	Intervention participants (*n* = 150) Control participants (*n* = 163)	Quantitative	Longitudinal	Developed specifically for study
Community Readiness Study	To determine the community’s readiness to change	Interviews with key stakeholders and randomly recruited community residents	Annual for 5 years	Stakeholders (*n* = 11) Residents (*n* = 9)	Qualitative – then scored to derive a quantitative score	Serial cross-sectional	Community Readiness tool (http://www.triethniccenter.colostate.edu/community-readiness-2/)
Resource Assessment study	To determine changes in quantity, and quality of healthy food and physical activity resources located in the community	Observations of all assets in the community using standardized protocol	Baseline and 5-year post test	Corner stores (*n* = 5) Community gardens (*n* = 2) Public parks (*n* = 5) Recreation center (*n* = 1)	Quantitative	Longitudinal	Developed specifically for this study

### Theoretical framework

The GAVA intervention and the evaluation protocol were developed using the socio-ecological model (SEM) framework, which proposes that individual behaviors are a result of different levels of influence [23]. These different levels of influence not only affect the individual’s behavior directly but also interact with each other. The levels comprise the intrapersonal level, which includes the individual factors (e.g., gender, age, self-efficacy) of the person who engages in the behavior; interpersonal factors (e.g., family support and social norms); the broader social environment (e.g., cultural norms, crime levels); and aspects of the built environment (e.g., availability and accessibility of grocery stores, parks, recreation facilities). The central basis of the GAVA initiative is to intervene at all of the different levels of the SEM with an emphasis on the built community environmental level.

### Selection of study community

Although the City of Austin is considered one of the healthiest cities in the U.S., Austin also has neighborhoods in which obesity and food insecurity prevalence rates are very high [4, 24]. The Dove Springs community is a predominantly Hispanic, low-income community that typifies a highly obesogenic environment, with some of the highest rates of obesity in Austin [25]. At the time of the start of this study, the community faced multiple barriers related to engaging in healthy eating and physical activity, including the lack of an easily accessible, full-service grocery; lack of safe, public recreation facilities; and programs that are too expensive for community members. This particular community was chosen as the intervention community for the GAVA initiative for reasons that are both positive and negative, based on the initial substudies (Community Readiness and Resource Assessment). These included: a high prevalence rate of obesity and food insecurity rates; (2) the presence of potential resources, such as green spaces, that could be developed; and (3) its active group of community stakeholders. Community Readiness interviews with community stakeholders in 2012 found that access to healthy foods and safe spaces to be physically active in the Dove Springs community were limited.

### Intervention approach

The GAVA initiative sought to address Dove Springs’ obesogenic environment by increasing the quality of existing resources and adding new resources to increase access to healthy food and physical activity opportunities. To ensure sustainability, the GAVA initiative was designed to build community capacity through the engagement of a large, community-wide coalition and the formation of resident teams at the start of the initiative. Organizational partners were brought in as content experts and, through GAVA, received capacity-building support and training to adapt their existing programming to make it more accessible to residents. Through the coalition, teams of residents organized around common interests related to four sectors: community food, community physical activity, school, and early childhood. To start a new team, residents created action plans, drawing from a list of “gold standard strategies” (i.e., evidence-based intervention strategies) and prioritized how they would address the issues specific to their own geographic area. Action plans are reviewed every quarter, and a combination of team and sector-wide meetings occur on at least a monthly basis to engage with residents regarding both the success and challenges of strategy implementation. From there, GAVA organizers work with community members and stakeholders to determine actionable next steps. Since the start of GAVA in 2013, approximately 30 formal resident teams within the GAVA Dove Springs Coalition have formed. Table 2 provides detailed information about the GAVA initiative, including the strategies chosen by resident teams for each of the four sectors.

**Table 2:** Resident strategies within four GAVA sectors at baseline

Outcome Category	Strategy Examples	Community Resources	Examples of Community Partners
Community Nutrition Sector: Increase easy access (geographic and financial) to healthy food			
Access	Identify and engage one or more small or medium sized local food stores for food access in the community.^1,2^	Local corner stores, farm stands, community gardens, public housing	Sustainable Food Center, The Food Trust, Foundation Communities, City of Austin Public Health Department
Access	Provide food stands in high density residential areas and public sites that promote access to fresh fruits and vegetables.^3^		
Utilization	Drive traffic to store and community food stands with events, celebrations, and promotions (ex, Food demonstrations with recipes, Buy one, Get one.., Frequent Buyer punch cards).^4,5^		
Sustainability	Create a resident-led team who will adopt a corner store and work with the store owner for the long term.^1,6^		
Physical Activity Sector: Increase easy access to safe physical activity facilities and programs			
Access	Improve and activate local parks, playgrounds, and recreation facilities: Ensure parks, playgrounds, trails, and recreation facilities are safe, well lit, maintained, and accessible.^7,8^	Local parks/pocket parks, creeks, greenbelts, streets	Austin Parks Foundation, Foundation Communities, City of Austin Parks And Recreation Department, Neighborhood Partnering Program, Austin Interfaith, Police Activity League, Austin Tenants’ Council
Utilization	Promote and support general park programming such as Movies in the Park, farmers markets, and other events that positively activate the park.^9,10^		
Utilization	Where appropriate, ensure functioning street lamps and park lights that create pleasant light throughout the night.^11,12,13^		
Sustainability	Ensure that park adoption team and neighbors support adequate investment and attention to parks from public officials and local leadership.^14^		
School Sector: Provide children with an environment that promotes healthy eating and physical activity			
Access	Improve and support enforcement of the district nutrition policy including cafeteria, a la carte, fundraising, vending, and food and beverage options at all events.^15^	Local elementary and middle schools, school gardens	Safe Routes to School, UT School of Public Health/Dell Center for Healthy Living, CATCH Global Foundation, Austin Independent School District, Afterschool Centers on Education (ACE)-Austin, The Austin Project
Utilization	Integrate physical activity into the day using strategies like Active Play/Fuel Up to Play 60, WOW Time, daily recess breaks and brain/activity breaks such as GoNoodle or HOPSport.^16^		
Utilization	Support supervised open gym and other physical activity opportunities in the morning, during lunch, and/or during out of school time.^17^		
Sustainability	Ensure that there is a single Coordinated School Health (CSH)/Wellness Team on campus that is generating, promoting and facilitating campus wide wellness/CSH activities.^18,19^		
Early Childhood Sector: Provide pre-school aged children with an environment that promotes healthy eating and physical activity			
Access	Encourage participation in food assistance programs such as WIC and SNAP.^20,21,22^	Early Development Centers, pre-K and K elementary classrooms	United Way for Greater Austin, Austin Independent School District, Austin Community College, Child Inc., Any Baby Can, Children in Nature Collaborative of Austin (CINCA)
Utilization	Encourage staff and parents to model healthy eating and physical activity behaviors.^23^		
Utilization	Promote classroom-based nutrition information such as the CATCH early childhood curriculum.^24^		
Utilization	Integrate opportunities for physical activity into the daily routine (movement activities).^25^		

### Evaluation approach

The purpose of the GAVA evaluation was to (1) assess both the short-term and long-term impact of the GAVA initiative on outcome variables and (2) monitor the ongoing implementation of the GAVA initiative as a means to provide feedback to the GAVA Implementation Team. Given that each resident team was able to choose different strategies and intervention targets, it was imperative to create a complex, multi-level evaluation plan that was both comprehensive and sensitive to change.

### Cohort study

The purpose of the Cohort Study was to examine the impact of the GAVA strategies on changes in key outcome variables related to awareness, use of community resources, and physical activity and healthy eating behaviors of residents who live in the GAVA intervention community as well as to compare these outcomes to residents who live in control communities. Our hypotheses stated that residents in the intervention group, compared to residents in the control group, would be more aware of, perceive fewer barriers to, and would make greater use of the physical activity and healthy eating resources in their community after 5 years of GAVA implementation. In addition, it was hypothesized that residents in the intervention group would be more physically active, engage in more healthy food behaviors, and have healthier body mass index (BMI) scores.

For the Cohort Study, 313 child-parent dyads (i.e., 150 dyads in the intervention community and 163 dyads in the control communities) were recruited and followed over a 5-year period. The intervention community was a single zip code which constituted the area covered by the GAVA intervention, while the control community consisted of communities that were similar to the intervention community in terms of demographics and access to healthy food and physical activity opportunities but that were geographically removed from the area of intervention. The intervention dyads were recruited from five Austin Independent School District’s (AISD) elementary schools located in the intervention community, while control dyads were recruited from five AISD elementary schools located in the control communities. Location information (i.e., street addresses) for each individual and dyad was collected to allow the mapping of participants, using a geographic information system (GIS), and to evaluate the impact of specific built environmental changes on individuals who lived in close proximity.

Consent from AISD and principals of all 10 schools was obtained prior to recruitment. Enrollment of parents and their kindergarteners into the study took place in all 10 schools during the 2013–2014 school year, starting with each school’s “Back to School” and “Meet the Teacher” events in August 2013. Researchers were allowed to set up a table and provide study information to all interested parents. Researchers also visited a few more school events until reaching the recruitment target number in October 2013. Inclusion criteria for the adults who participated in the Cohort Study were: parent or legal guardian of a kindergartener, 18 years or older, and literacy level sufficient to read and understand third grade English or Spanish. The inclusion criterion for the children was: enrolled in kindergarten at one of the study schools. All adult participants completed a yearly self-administered survey and were measured for height and weight at the start of each school year. Child participants’ height and weight also were measured.

For the Cohort Study, we planned an initial data collection of 300 parent-child dyads (150 each from the intervention and control areas) and anticipated an attrition of about 20% by the end of the 5-year follow up period. The resulting sample size of 240 child-parent dyads should have ample power (87 and 80%, respectively) to detect within-group changes in outcomes between time points as small as 0.18 standard deviation units, and differences in final outcomes between the study arms as small as 0.20 standard deviation units, with a threshold for detection set at alpha = .05. In terms of the outcomes of BMI *z*-score changes, BMI *z*-scores in young children are normally distributed with a mean and standard deviation of 0.34 and 1.5, respectively. With a sample size of 240, should the intervention achieve a difference of at least 0.3 BMI *z*-score units (equivalent to 0.18 standard deviation [SD] units) by the end of 5 years in the intervention group, we had a high probability of detecting that change as significant. The power of the design derives from the longitudinal design and the unusually large number of repeated measurements (five measurements, including baseline).

### Measures

The key outcome variables for this study included predictor variables of resource usage (i.e., awareness and perceived barriers), actual usage of resource, behavioral variables, and weight status. Specifically, the outcome variables included: perceived availability of safe and affordable physical activity (PA) resources, perceived barriers to using PA resources, utilization of PA resources, PA behaviors, awareness of availability of healthy food resources, barriers to fruit and vegetable (F&V) purchasing, utilization of healthy food resources, F&V availability at home, and healthy eating behaviors. BMI was calculated from measured heights and weights. An extensive set of socio-demographic variables also was included in the Cohort Survey.

Due to the importance of providing specific data to the GAVA implementation team, most items on the Cohort Survey were developed specifically for this study. Items and their response options are presented in Tables 3–6. The survey was translated in Spanish and pilot tested for cultural competence and appropriate literacy level before use in the field. The cohort survey took between 30 and 45 min to complete, and parents received monetary compensation for completion of the measures.

**Table 3:** Adult Sociodemographics by Treatment Condition at baseline (*N* = 313)

Variable	Total^a^ (*n* = 313)	Intervention^a^ (*n* = 150)	Control^a^ (*n* = 163)	*p*-value^b^
Gender				
Male	27 (8.6)	12 (8.0)	15 (9.2)	
Female	286 (91.4)	138 (92.0)	148 (90.8)	0.71
Ethnicity/Race				
Hispanic	271 (86.6)	133 (88.7)	138 (84.7)	
Black	20 (6.4)	9 (6.0)	11 (6.8)	
White	10 (3.2)	3 (2.0)	7 (4.3)	
Other	12 (3.8)	5 (3.3)	7 (4.3)	0.64
Language at home				
Primarily English	122 (40.3)	54 (37.0)	68 (43.3)	
Primarily Spanish	131 (43.2)	66 (45.2)	65 (41.4)	
Mix	50 (16.5)	26 (17.8)	24 (15.3)	0.52
Annual household income				
Under $25,000	226 (76.6)	106 (75.7)	120 (77.4)	
$25,001–$35,000	40 (13.6)	22 (15.7)	18 (11.6)	
$35,001–$50,000	17 (5.8)	8 (5.7)	9 (5.8)	
> $50,000	12 (4.1)	4 (2.9)	8 (5.2)	0.59
Marital status				
Never married	61 (20.3)	23 (16.2)	38 (24.1)	
Married	197 (65.7)	102 (71.8)	95 (60.1)	
Div/sep/wid	42 (14.0)	17 (12.0)	25 (15.8)	0.10
Number of children at home				
1–2	149 (48.5)	66 (44.3)	83 (52.5)	
3–4	130 (42.4)	67 (45.0)	63 (39.9)	
> 4	28 (9.1)	16 (10.7)	12 (7.6)	0.31
Number of adults at home				
1	61 (19.7)	28 (19.3)	33 (21.3)	
2	157 (50.7)	78 (53.8)	79 (41.0)	
> 2	82 (27.3)	39 (26.9)	43 (27.7)	0.87
Enrollment in federal food assistance (SNAP)				
No	162 (51.8)	71 (47.3)	91 (55.8)	
Yes	151 (48.2)	79 (52.7)	72 (44.2)	0.13
Food insecurity				
Almost never/never	128 (42.2)	60 (41.7)	68 (42.8)	
Sometimes	140 (46.2)	61 (42.4)	79 (49.7)	
Almost always/always	35 (11.6)	23 (16.0)	12 (7.6)	0.06

a. Totals for individual variables are less than group totals due to missing values; percentages do not always add up to 100 due to rounding errors

b. *p*-values for χ^2^ tests, *α* = 0.05

**Table 4:** Adult-perceived Availability, Barriers, and Utilization of Physical Activity Resources at baseline (*N* = 313)

Question	Total^a^ (*n* = 313)	Intervention^a^ (*n* = 150)	Control^a^ (*n* = 163)	*p*-value^b^
Perceived Availability of PA Resources				
Does your neighborhood have free or low-cost public recreation facilities?				
No	44 (14.3)	16 (11.0)	28 (17.3)	
Yes	212 (69.1)	102 (70.3)	110 (67.9)	
Don’t know	51 (16.6)	27 (18.2)	24 (14.8)	0.24
Does your neighborhood have enough free or low-cost public programs for physical activity?				
No	74 (24.4)	29 (20.4)	45 (28.0)	
Yes	92 (30.4)	42 (29.6)	50 (31.1)	
Don’t know	137 (45.2)	71 (50.0)	66 (41.0)	0.21
Does your neighborhood have playgrounds that are of good quality and safe?				
No	67 (22.0)	32 (22.2)	35 (21.9)	
Yes	197 (64.8)	89 (61.8)	108 (67.5)	
Don’t know	40 (13.2)	23 (16.0)	17 (10.6)	0.36
Is there a place to get drinking water when you are outside?				
No	154 (50.2)	84 (58.3)	70 (42.9)	
Yes	95 (30.9)	30 (20.8)	65 (39.9)	
Don’t know	58 (18.9)	30 (20.8)	28 (17.2)	< 0.001
Barriers to PA Resources				
% reporting that distance to facilities is a barrier to physical activity	23 (19.0)	8 (14.8)	15 (22.4)	0.19
% reporting that poor quality facilities is a barrier to physical activity	22 (18.2)	12 (22.2)	10 (14.9)	0.52
% reporting that safety of facilities is a barrier to physical activity	24 (19.8)	11 (20.4)	13 (19.4)	0.83
% reporting at least one barrier to physical activity	52 (43.0)	23 (42.6)	29 (43.3)	0.56
Utilization of PA Resources				
In the past 6 months, how often have you used the recreation center in your neighborhood for physical activity?				
Less than once a month	141 (48.8)	70 (50.0)	71 (47.7)	
1–2 times a month	25 (8.7)	17 (12.1)	8 (5.4)	
1–2 times a week	70 (24.2)	32 (22.9)	38 (25.5)	
3 or more times a week	53 (18.3)	21 (15.0)	32 (21.5)	0.12
In the past 6 months, how often have you used the neighborhood trails or streets for walking?				
Less than once a month	76 (25.5)	39 (27.9)	37 (23.4)	
1–2 times a month	29 (9.7)	17 (12.1)	12 (7.6)	
1–2 times a week	88 (29.5)	41 (30.0)	46 (29.1)	
3 or more times a week	105 (35.2)	42 (30.2)	63 (39.9)	0.24
In the past year, how often have you used the parks in your neighborhood for physical activity?				
Less than once a month	84 (27.8)	46 (32.2)	38 (23.9)	
1–2 times a month	33 (10.9)	21 (14.7)	12 (7.5)	
1–2 times a week	107 (35.4)	44 (30.8)	63 (39.6)	
3 or more times a week	78 (25.8)	32 (22.4)	46 (28.9)	0.04
In the past 6 months, how often have you used the playgrounds in your neighborhood?				
Less than once a month	61 (20.3)	28 (19.7)	33 (20.8)	
1–2 times a month	44 (14.6)	32 (2.5)	12 (7.5)	
1–2 times a week	107 (35.4)	51 (35.9)	56 (35.2)	
3 or more times a week	89 (29.6)	31 (21.8)	58 (36.5)	0.001

a. Totals for individual variables are less than group totals due to missing values; percentages do not always add up to 100 due to rounding errors

b. *p*-values for χ^2^ tests, *α* = 0.05

**Table 5:** Adult-perceived Awareness, Barriers to Fruits and Vegetables (F&V) Purchasing, and Utilization of Health Food Resources at baseline (*N* = 313)

Question	Total^a^ (*n* = 313)	Intervention^a^ (*n* = 150)	Control^a^ (*n* = 163)	*p*-value^b^
Awareness of Availability of Healthy Food Resources				
I don’t know whether there is a farmers market or farm stand in my community	139 (30.8)	72 (31.9)	67 (29.8)	0.22
I don’t know whether there is a mobile vegetable market in my community.	191 (42.4)	90 (39.8)	101 (44.9)	0.72
I don’t know whether there is a community garden in my community	121 (26.8)	64 (28.3)	57 (25.3)	0.16
Barriers to F&V Purchasing				
Have you had any of the following issues when you buy fruits and vegetables for your family?				
The fruits and vegetables are of low quality	59 (21.1)	30 (20.4)	30 (21.9)	0.83
Poor selection of fruits and vegetables	74 (26.5)	40 (28.2)	34 (24.8)	0.23
The fruits and vegetables are expensive	93 (33.3)	49 (34.5)	44 (32.1)	0.27
Not available in stores where I buy food	13 (4.7)	4 (2.8)	9 (6.6)	0.21
Other Reason	40 (14.3)	20 (14.1)	20 (14.6)	0.78
Average number of barriers to F&V purchasing				
Mean (SD)	2.27 (1.87)	2.35 (1.85)	2.19 (1.90)	0.45
Utilization of Healthy Food Resources				
Where do you obtain fruits and vegetables for your family? (Check all that apply)				
Supermarket	301 (96.2)	157 (96.3)	144 (96.0)	
Other	42 (3.4)	22 (14.7)	20 (12.3)	0.88
Do you use a farmers market or farm stand in your community?				
Rarely or never	253 (83.8)	126 (86.3)	127 (81.4)	
Yes, regularly or sometimes	49 (16.2)	20 (13.7)	29 (18.6)	0.25
Do you use a mobile vegetable market in your community?				
Yes, regularly or sometimes	21 (17.2)	10 (16.7)	11 (17.7)	
Rarely or never	101 (82.8)	50 (83.3)	51 (82.3)	0.88
Do you use a community garden in your community?				
Yes, regularly or sometimes	10 (5.2)	7 (8.1)	3 (2.8)	
Rarely or never	182 (94.8)	79 (91.9)	103 (97.2)	0.10

a. Totals for individual variables are less than group totals due to missing values; percentages do not always add up to 100 due to rounding errors

b. *p*-values for χ^2^ tests, *α* = 0.05

**Table 6:** Adult Physical Activity, Healthy Eating Behavioral Outcomes, and BMI at baseline (*N* = 313)

Question	Total^a^ (*n* = 313)	Intervention^a^ (*n* = 150)	Control^a^ (*n* = 163)	*p*-value^b^
Physical Activity Outcomes				
During the past 7 days, how many times did you exercise or take part in any vigorous physical activity for at least 20 min?				
Never	56 (18.9)	33 (21.0)	23 (16.5)	
1–2 times	106 (35.8)	49 (31.2)	57 (41.0)	
3–4 times	90 (30.4)	49 (31.2)	41 (29.5)	
5–6 times	27 (9.1)	17 (10.8)	10 (7.2)	
7 times	17 (5.7)	9 (5.7)	8 (5.8)	0.50
During the past 7 days, did you walk for exercise (outdoor, indoor, treadmill)?				
Yes	214 (68.4)	105 (70.0)	109 (66.9)	
No	99 (31.6)	45 (30.0)	54 (33.1)	0.55
Healthy Eating Outcomes				
How often do you have fresh fruits and vegetables available in your home?				
Never	2 (0.7)	1 (0.6)	1 (0.6)	
Rarely	16 (5.3)	8 (4.9)	8 (4.9)	
Sometimes	127 (41.9)	58 (41.1)	69 (42.6)	
Often	36 (11.9)	17 (12.1)	19 (11.7)	
Always	122 (40.3)	57 (40.4)	65 (40.1)	0.99
How often do you have frozen, dried, or canned fruits or vegetables available in your home?				
Never	30 (9.8)	14 (9.8)	16 (9.9)	
Rarely	51 (16.7)	25 (17.5)	26 (16.0)	
Sometimes	78 (25.6)	32 (22.4)	46 (28.4)	
Often	85 (27.9)	41 (28.7)	44 (27.2)	
Always	61 (20.0)	31 (21.7)	30 (18.5)	0.81
Do you eat more than one variety of fruit each day?				
No	32 (10.2)	12 (8.0)	20 (12.3)	
Sometimes	160 (51.1)	77 (51.3)	83 (50.9)	
Yes, often	78 (24.9)	41 (27.3)	37 (22.7)	
Yes, every day	43 (13.7)	20 (13.3)	23 (14.1)	0.55
Do you eat more than one variety of vegetables each day?				
No	29 (9.4)	13 (8.8)	16 (9.9)	
Sometimes	280 (90.3)	134 (90.5)	146 (90.1)	
Yes, often	0 (0.0)	0 (0.0)	0 (0.00)	
Yes, every day	1 (0.6)	1 (0.6)	0 (0.00)	0.68
What is the total amount of fruit you eat each day?				
0 cups	13 (4.2)	5 (3.4)	8 (5.0)	
½ cup	65 (21.2)	30 (20.5)	35 (21.7)	
1 cup	120 (39.1)	59 (40.4)	61 (37.9)	
1-½ cups	63 (20.5)	31 (21.2)	33 (19.9)	
2 cups	46 (15.0)	21 (14.4)	25 (15.5)	0.95
What is the total amount of vegetables you eat each day?				
0 cups	11 (3.6)	3 (2.1)	8 (5.0)	
½ cup	85 (27.7)	46 (31.5)	39 (24.2)	
1 cup	98 (31.9)	39 (26.7)	59 (36.6)	
1-½ cups	66 (21.5)	38 (26.0)	28 (17.4)	
2 cups	47 (15.3)	20 (13.7)	27 (16.8)	0.07
BMI Categories (Measured)				
Parent				
Normal weight	59 (19.0)	28 (19.0)	31 (19.0)	
Overweight	86 (27.7)	46 (31.3)	40 24.5)	
Obese	165 (53.2)	73 (49.7)	92 (56.4)	0.38
Child				
Normal weight	187 (60.1)	93 (62.4)	94 (58.0)	
Overweight	40 (12.9)	20 (13.4)	20 (12.3)	
Obese	84 (27.0)	36 (24.2)	48 (29.6)	0.56

a. Totals for individual variables are less than group totals due to missing values; percentages do not always add up to 100 due to rounding errors

b. *p*-values for χ^2^ tests, *α* = 0.05

#### Body mass index

Trained staff used standard equipment (digital scale and stadiometer) and calibration procedures to measure body weight to the nearest 0.1 kg and height to the nearest 1 mm, as described by the National Center for Health Statistics [26]. Each measure was taken twice, and the average was recorded. For adults, heights and weights were used to calculate BMI for each participant, using the equation kg/m^2^. Adults with a BMI of 18.5–24.9 were classified as normal weight, those with a BMI of 25–25.9 were classified as overweight, and those adults with a BMI of 30 or greater were classified as obese [27]. For the children, BMI (weight [kg]/ stature [m2]) z-score for age and sex was computed using the 2000 Centers for Disease Control and Prevention reference, which is adjusted for age and gender. Children with a BMI percentile score in the 5th–84th percentiles were classified as healthy weight, children who are in the 85th–95th percentiles were classified as overweight, and those over the 95th percentile, as obese [28].

#### Demographic data

Items on the demographic portion of the survey included gender, ethnicity/race, language spoken at home, household income level, marital status, number of children and adults at home, receipt of Supplemental Nutrition Assistance Program (SNAP) benefits, and food insecurity. All items were previously used in other studies with similar populations (Table 3) [29, 30].

### Outcome data

*Perceived availability of resources*. *Perceived availability of safe and affordable PA resources* (e.g. recreation center, public programs, playgrounds) was measured with three items that had “no/yes” responses. A separate question asked about the availability of water.

*Awareness of availability of healthy food resources* was measured with three items that assessed awareness of farm stands, healthy corner stores, and mobile markets.

*Barriers to access*. *Barriers to PA access* was measured with three items that assessed the barriers of distance, poor quality, and safety. “Percentage reporting at least one barrier” was assessed by summing the number of barriers that each individual reported.

*Barriers to purchasing F&V* was measured with one question: “Have you had any of the following issues when you buy fruits and vegetables for your family?” The following responses were offered: F&V are of low quality, poor selection of F&V, F&V are expensive, F&V not available at the stores where I shop, and “other reasons.” Participants could select more than one answer. “Average number of healthy eating barriers” was assessed by summing and averaging the number of barriers each individual reported.

*Utilization of resources*. Three items were used to measure frequency of *utilization of physical activity resources* in the community, including the recreation center, trails/streets, parks, and playgrounds. Item responses ranged from less than once a month, 1–2 times a month, 1–2 times a week, or 3 or more times per week.

*Utilization of healthy food resources*, including supermarkets and alternative retail options (i.e., farm stands or mobile markets) was measured using four items. The item for grocery store usage asked where the participants obtain F&V. The supermarket was one of the options. For the other three items, participants were specifically asked how often they had used the alternative retail options. The response options included never, rarely, sometimes, or regularly.

*Behavioral variables*. Time spent in vigorous activity and walking were assessed through two items. For vigorous activity, participants were asked how often they exercised or took part in vigorous activity for at least 20 min during the past 7 days. The responses ranged from never to 7 times. To measure time spent in walking, the respondent was asked whether he/she walked during the past 7 days. Responses were either “yes” or “no.” This item was part of a validated instrument [31].

*Healthy eating behaviors* were measured using five items. These items assessed the amount of fruit consumed each day, amount of vegetables consumed each day, consumption of F&V as snacks, and variety of F&V consumed. Home availability of F&V was measured with two items on the availability of fresh F&V and frozen/dried/or canned F&V. These two items were adapted from a validated instrument [32].

### Data analysis

Analyses presented in this paper are primarily simple two-way tabulations of variables across treatment conditions, without adjustment for other possible confounders. Chi-square tests were utilized to examine differences in distribution of levels of the outcome variables across the study cohort and the control group participants. *P*-values corresponding to the chi-square values are presented to assess the extent to which the study group differed from the control group. A threshold value of 0.05 was utilized to conclude if there was a statistically significant difference in distribution of outcome variables across the study and control groups. All data analyses were conducted after extensive data cleaning, which consisted of replacing implausible values with missing values.

## Results

At baseline, 313 parent-child dyads were recruited for the Cohort Study. Parents’ sociodemographics, according to the study group, are presented in Table 3. Cohort respondents were mainly Hispanic women. The language spoken at home was evenly divided between English and Spanish. Household income levels were low, with 77% as earning less than $25,000 per year. Even though approximately 50% of respondents were enrolled in SNAP benefits, food insecurity was high, with 58% of participants’ responding that they were *sometimes* or *almost always* food insecure. The control group reported significantly higher rates of *always* having food insecurity, while the intervention group reported significantly higher enrollment in SNAP.

Data related to physical activity resources availability, barriers, and utilization are presented in Table 4. Overall, perception of availability of physical activity resources was fairly high, with most individuals’ being aware of public recreation facilities (69%) and safe playgrounds (65%). However, 70% of respondents felt that there were not enough or they did not know of any low-cost public programs. Some differences were noted between the intervention and control groups, with intervention participants’ being less aware or responding “no” to the question about availability of enough public programs. In addition, compared to the control participants, more intervention participants responded that there were no places to get drinking water in their community. In terms of barriers to physical activity, all three specific barriers (i.e., distance, poor quality of the resource, and safety) were mentioned by approximately 8% of the respondents. Overall, 43% of all respondents mentioned at least one barrier. No significant differences were found between the intervention and control groups. In terms of utilization of PA resources, the public recreation center was the resource least often used (18% reported using it 3 or more times per week). Streets/trails were the most often used resource (approximately 35% reported using them 3 or more times per week). Control parents reported using the playgrounds significantly more often than did the parents in the intervention group, while intervention parents reported using the parks more often.

Data related to healthy food resources availability, barriers, and utilization are presented in Table 5. A large percentage of the respondents indicated not being aware of alternative healthy food resources, such as farmers markets, mobile vegetable markets, or community gardens, in their community. This perception was correct in that, at the time of the baseline measurement, in both communities, there were no farmers markets or mobile markets and only two community gardens. Most participants (96%) reported using a supermarket to obtain their F&V. However, barriers to purchasing high-quality and fresh F&V were reported. The most common barrier to purchasing F&V was the expense (33%), followed by poor selection of F&V (27%) and low quality F&V (21%). On average, participants reported 2.27 barriers to purchasing F&V. Very few participants used alternative healthy food resources, with farmers markets as the one alternative resource used most often (16%). No significant differences were noted between intervention and control participants for any of the variables related to healthy food resources.

Physical activity and healthy eating behaviors are reported in Table 6. Of the participants, 45% reported being vigorously active more than twice a week, and 32% reported not walking at all during the past week. In contrast, in terms of healthy eating, participants reported consuming F&V on a regular basis. Of the participants, 52% reported having fresh F&V available in their homes *often* or *always*, while 48% reported consuming F&V as snacks *often* or *every day* (data not shown). Participants did not necessarily eat a large variety of F&V. In terms of fruit, 38% of participants reported eating a variety of fruits *often* or *each day*. In terms of vegetables, fewer than 1% of respondents reported eating a variety *often* or *every day*. Participants consumed more fruits than vegetables. Approximately 75% reported eating 1 cup or more of fruit per day, and 69% reported eating 1 or more cups of vegetables per day. No significant differences were noted between intervention and control participants.

Both parent and child participants showed a very high prevalence of overweight and obesity. Among adults, 53% of participants were classified as obese and 28% as overweight. Among children, 27% were classified as obese and 13% as overweight. No differences were found between the intervention and control group participants related to overweight and obesity classifications.

## Discussion

The purpose of this paper was to describe the GAVA evaluation plan and to present selected baseline data obtained through the cohort sub-study. The GAVA initiative targeted low-income, predominantly Hispanic communities located in an urban area in central Texas. The particular communities were chosen due to several unique characteristics, which, when taken into consideration simultaneously, provided the perfect opportunity for testing an innovative place-based initiative. These characteristics included high need due to a high prevalence of obesity and related chronic diseases, high rates of food insecurity (which, although not correlated directly with poverty, can be used as a proxy for poverty), presence of potential environmental resources (e.g., green spaces that were underdeveloped), and community key stakeholders who were willing to engage.

The sociodemographic results from the baseline cohort data collection confirm that the families enrolled in the study will likely benefit from efforts focused on obesity prevention. In the intervention community, obesity rates among adults and children were 54 and 27%, respectively, which is substantially higher than national averages [33]. In addition, this population also reported high food insecurity rates, with 46% who reported being *sometimes* food insecure and 12% who reported *always* being food insecure. Again, compared to national data that indicated a 12.3% food insecurity rate, our participants were substantially more food insecure [34].

Behavioral data indicated that many participants did not engage in regular physical activity. Approximately 32% of participants reported no walking for exercise during the past 7 days. Given that walking is the most common physical activity for adults [35], increases in this variable will be indicative of more physical activity in this population group. Participants reported relatively high rates of F&V consumption, which is consistent with other studies that indicate that Hispanic individuals tend to have relatively high rates of F&V consumption [36].

The results from our Resource Assessment Study (data not shown in this manuscript) show that, at baseline, there was a lack of appropriate and attractive green spaces in the intervention community. Although the built environment of the community did include green spaces, such as parks and a public recreation center, many of the green spaces were not kept up and were not considered safe or attractive. Consistent with these results are relatively low rates of utilization of green spaces, with 38% of individuals reporting using parks or playgrounds only two times per month or less. About 42% of individuals, however, did report using the public recreation center at least once a week, indicating that this resource could be a good location for the implementation of future physical activity programming or for other community programs, such as food stands or food delivery. Other studies indicate the use of recreation centers as hubs for other healthy living activities, such as adult education training programs that include the learning of time management skills and that provide a sense of belonging [37].

In terms of access to healthy food, the only retailer for which individuals had access to healthy foods was a grocery store, which is located next to a major freeway and difficult to reach by foot for all participants residing a mile from the freeway. Although most participants did report shopping at this particular grocery store for F&V, participants reported barriers such as cost, poor selection, and low quality of F&V. Other studies have found these same barriers in other low-income communities. Even in low-income communities with a grocery store, the stores tend to provide lower quality and sometimes higher priced F&V [38]. Utilization of alternative options to obtain fresh, quality produce, such as farmers markets, mobile vegetable markets, or community gardens, was very low, which was not surprising, as this community did not have these options.

The demographic breakdown of the GAVA participants (mostly low-income and Hispanic) indicated the need for some special considerations related to data collection procedures and intervention materials/strategies. Specifically, the intervention materials needed to address certain socioeconomic, home environment, and cultural issues that could potentially influence dietary and PA behaviors of the participants. In terms of evaluation, all study materials needed to be available in both English and Spanish, and during data collection, there was a need for bilingual, trained data collection staff. For this study, we were able to hire community residents, and it was found to be beneficial to enlist the assistance of community residents with the data-collection efforts.

### Limitations and strengths

As with any study, this study has some limitations. First, given the need to create a survey that was comprehensive enough to measure both physical activity- and healthy eating-related variables, but short enough to allow individuals with possibly low literacy levels to complete, it was necessary to measure most of our variables with only one or two items. This is not ideal, and it is possible that our measures are not sensitive enough to measure change at a community-wide level. This was, however, taken into account before the start of the study and the reason for multiple sub-studies within the GAVA evaluation protocol. In addition, due to the need for specific process data to feed back to the implementation team, items were developed specifically for the GAVA intervention. In many cases, this precluded the use of previously validated items or instruments. Second, because our participants were not randomly assigned to control or study conditions, but rather, drawn from a pre-determined intervention community and a posthoc control community, it was not possible to obtain probabilistically equivalent study and control groups at baseline. Under these conditions, it was inevitable that on some of the variables we examined, there were statistically significant differences between study conditions. However, given that the sociodemographics are largely similar, we believe that there is sufficient comparability across the study conditions.

Strengths of the GAVA study include its innovative approach to evaluation of community-based interventions. While community-led and geographically-based coalitions promise to be sustainable and culturally appropriate [39], detecting significant changes is difficult in a real-world setting since dosage is often low and strategies evolve over time. Conducted over 5 years, the GAVA evaluation is able to track and assess changes over time as the intervention responds to shifting community priorities. The GAVA evaluation employs a multimodal approach to capture various aspects of the intervention’s effect, using individual-level (Cohort Study), and environmental / social-level studies (Resources Assessment, Community Readiness). This allows assessment of the various impacts of a complex intervention. Finally, our study is unique in including measures relating to perceived access to and utilization of community resources, outcomes that are important in assessment of community-level, place-based interventions.

## Conclusion

The GAVA Initiative is an ongoing intervention that was created as a place-based, multicomponent obesity initiative designed to synergistically target multiple sectors and systems, with an emphasis on environmental aspects of the community. The overall goal of the GAVA initiative is to change the built environment to make it easier for residents to use community resources, which will lead to increased healthy eating and physical activity. Our evaluation plan includes several sub-studies, and at the end of the 5-year study period, data from the different sub-studies will be triangulated to provide a detailed picture of what exactly happened during the 5-year GAVA intervention. To date, we have completed our final round of data collection for the Cohort Study, but all other sub-studies are still underway. Preliminary, unpublished results indicate positive changes in the built environment of the GAVA intervention community. Results from the GAVA study will lead to the refinement of a model that is intended to be replicated in other locations across the U.S. Given the prevalence of unhealthy eating, insufficient physical activity and unhealthy weights among individuals in the U.S, as well as the limitations of interventions that focus primarily on individuals, more models, such as the GAVA initiative, are warranted.

## Abbreviations

BMI: Body mass index
F&V: Fruit and vegetable
GAVA: Go! Austin/Vamos! Austin
GIS: Geographic information system
PA: Physical activity
SD: Standard deviation
SEM: Socio-ecological model
SNAP: Supplemental Nutrition Assistance Program
U.S: United States
